# The relationship between sex steroids (E2, progesterone, and AMH) levels and severity and fatality of COVID-19: A systematic review

**DOI:** 10.1016/j.heliyon.2023.e14218

**Published:** 2023-03-01

**Authors:** Kowsar Qaderi, Hossein Hosseinirad, Mehri Kalhor, Sanaz Zangeneh, Marjaneh Pournaghi, Rasa Khodavirdilou, Maryam Keshavarz, Farideh Eghdampour, Seyedeh Tahereh Mirmolaei, Younes Jesmani, Samira Barjasteh, Manthar Ali Mallah, Ahmadreza Shamsabadi

**Affiliations:** a PhD in Reproductive Health, Midwifery Department, School of Nursing and Midwifery, Kermanshah University of Medical Sciences, Kermanshah, Iran; b Student Research Committee, School of Medicine, Shahid Beheshti University of Medical Sciences, Tehran, Iran; c PhD in Reproductive Health, Department of Midwifery, School of Nursing and Midwifery, Shahid Beheshti University of Medical Sciences, Tehran, Iran; d Midwifery and Reproductive Health Department, Student Research Committee, School of Nursing and Midwifery, Isfahan University of Medical Sciences, Isfahan, Iran; e Department of Reproductive Biology, Faculty of Advanced Medical Sciences, Tabriz University of Medical Sciences, Tabriz, Iran; f School of Nursing and Midwifery, Iran University of Medical Sciences, Tehran, Iran; g Department of Midwifery, Islamic Azad University, Marand, Iran; h Department of Midwifery and Reproductive Health, Nursing and Midwifery School, Tehran University of Medical Sciences, Tehran, Iran; i Kermanshah University of Medical Sciences, Kermanshah, Iran; j Reproductive Health Researcher Center, Clinical Research Center, Urmia University of Medical Sciences, Urmia, Iran; k College of Public Health, Zhengzhou University, 100 Kexue Ave, Zhongyuan District, Zhengzhou 450001, China; l Department of Health Information Technology, Esfarayen Faculty of Medical Science, Esfarayen, Iran

**Keywords:** Estrogen, Progesterone, Sex steroids, Female, Menopause, Corona virus, SARS-CoV-2, COVID-19

## Abstract

Sex steroids are powerful modulators of the immune system and they may affect the immune response and inflammatory consequences of COVID-19. This systematic review aims to explore the impact of sex steroids on COVID-19 mortality and complications. We looked up the keywords of the study in Scopus, PubMed, and Web of Science. All related original articles published in English, as of October 16, 2021, were reviewed to be included in our research. Concerns regarding the effect of sex hormones on COVID-19, eight full texts have been identified for the conclusion. In these studies, the relationship between estradiol and COVID-19 mortality has been mentioned. The most significant findings were the higher COVID-19 mortality rate in men, compared to women; also, in menopausal women compared to younger women and who received estradiol. In two studies, oral contraceptive pills had a protective effect on the morbidity of SARS-CoV-2 infection. In a randomized controlled trial, subcutaneous injection of progesterone in hospitalized men significantly reduced their symptoms and need for oxygen therapy. Hormone replacement therapy was positively associated with reducing COVID-19 symptoms. Although the results were insufficient for a conclusion, this study represents estrogen as an appropriate pharmacological method for preventing and diminishing the inflammation related to COVID-19 disease. However, future prospective studies and clinical trials are needed to clarify and approve this protective effect.

## 1 Introduction

Sex steroids are potent modulators of the immune system, and varying concentrations of estrogens and androgens between men and women may affect the immune response and inflammatory consequences of COVID-19 [1]. Since sex hormones are produced endogenously, maintaining normal levels ensures that they have no adverse effects [2]. Estrogens are sex steroid hormones and as such show an extensive range of physiological functions [3]. Experimental results indicate the involvement of sex steroid hormones in the Coronavirus disease outcomes, infection austerity, and fatality in women and men. Pinna proposed that "The severity of covid-19 symptoms and mortality is more prevalent in men than in women, indicating that progesterone and estrogen play a protective role in women." Estrogens may have anti-inflammatory effects during SARS-CoV-2 infection at various stages, ranging from increasing antiviral resistance in individual cells to suppressing pro-inflammatory cytokine production [4]. However, no dependable biochemical, physiological, or clinical proof has been presented to support this comprehensive and essential theoretical hypothesis [5]. Patients with COVID-19 have lower levels of E2 than control subjects, according to another study [6]. This issue could be remarkably important as acute and severe diseases, such as COVID-19, may change the hypothalamic-pituitary-gonadal axis function and reduce the endogenous creation of estrogens and testosterone [7]. The results of previous studies have shown that acute respiratory distress syndrome (ARDS), which is a pro-inflammatory condition that can lead to life-threatening pneumonia, has a direct effect on serum testosterone levels so that its level decreases during the early phase of the disease and continues to decline in the chronic phase. LH-independent testosterone appears to decrease, which may indicate an invalidism of the hypothalamic-pituitary-gonadal axis [8,9]. Slighter ARDS, a complexity of extreme sepsis, and sepsis-related fatalities in women than men were associated with high IL-6 levels and independent of age and illness [10].

According to some findings from ongoing clinical trials, estradiol could have a chance to treat severe Covid-19. Animal model studies are required to clarify the sex bias and the potential contribution of estradiol to Covid-19 morbidity and mortality [11]. Precise investigation of variations in steroid hormone levels during COVID-19 can shed light on the management and even treatment of this contagious disease, which was the purpose of this systematic review study.

## 2 Materials and methods

This systematic review of published articles, as of October 16, 2021, explored variations in steroid hormone levels during the COVID-19 pandemic. We looked up the keywords of the study in the database of Scopus, PubMed, and Web of Science. Also, we conducted hand searches from the reference lists of retrieved studies.

The search strategy for this research was organized in collaboration with three research team members. Key search terms include COVID-19, SARS-CoV-2, estrogen, progesterone, and genital hormone. The complete search strategy is as follows:

A: COVID-19 OR SARS-CoV-2 OR “Corona virus” OR Coronavirus OR COVID.

B: "genital hormone" OR estrogen OR progesterone OR "follicle-stimulating hormone" OR "Luteinizing hormone" OR "Gonadotropin-releasing hormone"

C: [A] AND [B]

Search results can be seen at Supplementary File 1. The EndNote software was used to organize studies recognized in the review. Search results from reviewed online databases are joined into a single EndNote library and removed duplicate records of the same reports. Two authors independently screen titles and abstracts of articles to determine if they meet the inclusion and exclusion criteria for selected articles.

Inclusion criteria:

- The original articles surveyed the impact of genital hormones on COVID-19.
- The articles published from the beginning of COVID-19 to October 16, 2021

Exclusion criteria:

− Non-original articles including case reports, reviews, editorials, and clinical trial protocol
− Non-full texts, abstract papers, short communication, and conference abstracts
− Non - English language

The table of data extraction includes the first author, country, study design, study population, reproductive hormones, the impact of hormones on COVID-19 outcomes, and other findings. Two authors independently extracted data, once studies are selected. To exclude any probable duplications, once again, the selected articles were surveyed by another researcher.

Reporting of this study was in accordance with the Preferred Reporting Items for Systematic Reviews and Meta-Analyses (PRISMA) checklist (8). The quality of the articles was surveyed by two independent and experienced authors, and any disagreement of opinion between two authors regarding the removal or selection of articles was resolved by a third author review and consensus.

### 2.1 Quality assessment

To assess the possibility of bias in the studies, we used the Newcastle-Ottawa scale (NOS) [12]. We used the Newcastle-Ottawa scale (NOS) to analyze the risk of bias in the studies [12]. For each study, NOS assigns a maximum score of 9 across the three categories of exposure, comparability, and selection. A score of 4 or less has been deemed "poor," and a score of 5 or more has been deemed "acceptable."

### 2.2 Ethical considerations

This article is based on published data, and hence ethical approval is not required.

## 3 Results

**Fig. 1:**
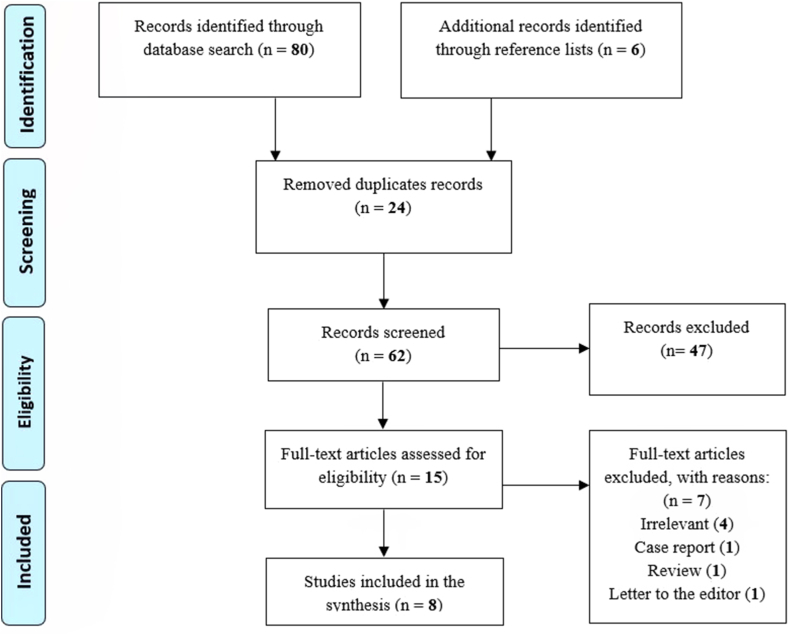
PRISMA flowchart.

A total of 86 related articles were identified through searches of online databases selected and manual searches. After removing duplicates, 62 articles remained, and then 47 articles were excluded after titles and abstracts screening, and the full texts of 15 residual articles were assessed for eligibility. Finally, eight articles, meeting the inclusion criteria, were included in this systematic review (Fig. 1). All studies received acceptable scores on the risk of bias evaluation (Table 2).

We extracted data from two retrospective [13,14], two cohorts [15,16], two cross-sectional [17,18], and two randomized controlled trials [19,20] studies. They have been conducted in India [14,17,19], China [16,18], the USA [20], the United Kingdom [15], and Germany [13] (Table 1).

**Table 1:** The protective role of sex steroids against COVID-19.

The first author Year (reference) Country	Study Design	Participant	Age	Past Medical History	Estrogens (E2), Progesterone, AMH	Hormones and COVID-19 outcomes (Severity of symptoms, and hospitalization)	Mortality due to COVID-19	Other Findings
Seth 2021 [19] India	randomized controlled trial	60 women 20: intervention 40: control	Intervention group: 61.1 ± 8.71 Control group: 62.425 ± 9.84	Hypertension, Diabetes mellitus, Heart disease, Thyroid disorder, Tuberculosis	**Intervention group:** estradiol valerate tablet 2 mg daily (7 days), and the standard care. **Control group:** standard care only.	1- Rate of reverse PCR negativization in the intervention vs control on day 5 and 7 of admission P = 0.007; P = 0.026). 2-No significant difference in the duration of hospitalization (P = 0.213). 3-A significant difference in the number of patients progressed to moderate (P = 0.057), the mean values of inflammatory biomarkers – D-dimer, LDH, CRP on day 5 in the intervention group. 4-Interleukin-6 also showed a declining trend on day 5 in the intervention group, while control arm had a rising trend.	There were no mortality or adverse events in either group.	Oral estradiol in postmenopausal females can be a novel and efficient option for managing non-severe COVID-19 infection.
Ghandehari 2021 [20] USA	a pilot, randomized controlled trial	42 men 22: control group 20: progesterone group	55/3 ± 16/4	COVID-19 patients (Hypertension, diabetes, obesity)	Progesterone 100 mg, twice daily addition to Standard Care	2- Patients treated with progesterone required three fewer days of supplemental oxygen. They were hospitalized for 2.5 fewer days, in comparison with control group.	–	–
Costeira 2021 [15] UK	Cohort	152,637 women COC pill: 295,68 HRT:151,193	Menopausal 53.8 Premenopausal 45.2	women with menopausal status	E2 in form of COC pills and HRT	COCP-users had reduction in **hospitalization** (P = 0.023). Predicted COVID-19 had higher rates in menopausal women (P = 0.003), and lower rates in COCP-users (P = 8.03E-05).	–	–
Ding 2021 [16] China	Cohort	1902 patients	42.45 ± 13 female:26%	Confirmed hospitalized COVID-19	E2 and AMH	Both AMH and E2 levels were adversely **correlated** with infection severity (Adjusted HR, 0.146 [95% CI, .026–.824], P = 0.029 and 0.304 [95% CI, .092–1.001], P = 0.05, respectively).	–	E2 had significant negative correlation with IL- 2R, IL-6, IL-8, and TNF alpha (P = 0.03, P = 0.04, P = 0.05, and P = 0.02)
Ding 2021 [18] China	Cross-Sectional	78 females	<50	COVID-19 patients	AMH	AMH was lower in covid compared with healthy age-matched participants (p = 0.003).		
Arya 2021 [17] India	Cross-sectional	65 women 27 menopauses 38 non-menopauses	Menopause: 49.74 ± 3.97 Non menopause: 43.37 ± 3.51	Patients with co-morbidities excluded	Estrogen	Statistically significant difference was between the mean D-dimer (p = 0.04), C-Reactive protein (p = 0.04), and the number of moderate-to-severe disease (p = 0.005) in the menopause and non-menopause group.	–	–
Seeland 2021 [13] Germany	Retrospective	37,086 women Of 18,892 aged 15–49 years, 2078 received estradiol. Of 16891 aged 50+, 439 received estradiol.	15–49 (pre-menopausal) 50+ (postmenopausal)	COVID-19 positive patients with supplemental estradiol versus subjects without estradiol	Estradiol (E2) hormone therapy or contraceptive	–	**Fatality** was **less** in women 50+ estradiol-user group vs. the non-user group. OR = 0.33 [0.18, 0.62] hazard ratio = 0.29 [0.11, 0.76] women 15–49 years: OR = 1.0 [0.41, 2.4] HZ = 0.28 [0.1–1.1]	Mortality rate in women over 50 treated with estradiol was **50% lower** than in women of the same age without hormone intake. The protective effect of taking oral contraceptive pills was less in younger women than in older women
Garg 2020 [14] India	Retrospective	720 patients Male: 427 females: 293 Premenopausal 185 Postmenopausal:108	58.30 (42–82) female:40%	Covid-19 positive (RT_PCR)	–	–	Mortality rate: **females**: 12.66% **males**: 19.4% **premenopausal** females: 8.6% **postmenopausal** females:19.4%	Mortality was greater in males compared to females, and in postmenopausal females compared to premenopausal females.

**Table 2:** Newcastle–Ottawa scale quality assessment for the included studies.

ID	First author	Selection (Out of 4)	Comparability (Out of 2)	Outcome (Out of 3)	Total (Out of 9)
1	Seth 2021	***	***	***	7
2	Ghandehari 2021	***	***	***	7
3	Costeira 2021	***	***	***	8
4	Ding 2021 Cohort	***	***	***	8
5	Ding 2021	***	***	***	6
6	Arya 2021	***	***	***	6
7	Seeland 2021	***	***	***	9
8	Garg 2020	***	***	***	7

Some studies have examined the role of sex hormones in the incidence and complications of COVID-19 disease. In a retrospective analysis, COVID-19 mortality was greater in males compared to females (19.4% vs. 8.6%) and in the postmenopausal age group than in younger females (19.4% vs. 8.6%, respectively) [14].

In a pilot, randomized controlled trial, subcutaneous injection of 100 mg of progesterone twice daily in hospitalized men with moderate to severe hypoxic situations caused a reduction in symptoms and need for oxygen therapy. No serious adverse events attributable to the injected progesterone were reported [20].

The mortality rate of women over 50 who had been treated with supplemental estradiol (E2) was 50% lower than women of the same age without hormone intake [13]. Taking E2 has also been investigated in two other studies. E2 in the form of COC pills (combined oral contraceptive) significantly decreased hospital attendance (P = 0.023) [15]. Hormone replacement therapy (HRT) was positively related to reducing COVID-19 symptoms [15]. Estradiol and anti-mullerian hormone (AMH) levels had a significant inverse relationship with the COVID infection severity in women of reproductive age [16,18].

An RCT, comparing the effect of taking estradiol valerate tablet 2 mg daily for 7 days with the standard care, demonstrated that the rate of reverse PCR negativization in the intervention group at days 5 and 7 of admission P = 0.007; P = 0.026) was significantly higher than the control group. Also, a significant difference in the number of patients who progressed to moderate (P = 0.057) was observed even though no significant difference was found in the duration of hospitalization (P = 0.213). The intervention group had the lower mean values of inflammatory biomarkers – D dimer, LDH, and CRP on day 5 compared with the control group. Interleukin 6 also showed a declining trend on day 5 in the intervention group, while a control arm had a rising trend [19].

Statistically, a significant difference was between the mean D-dimer (p = 0.04), C-Reactive protein (p = 0.04), and the number of moderate-to-severe disease (p = 0.005) in the menopause and non-menopause group [17].

## 4 Discussion

The obtained results from this study verified that women are less vulnerable to COVID-19 infection than men and the prevalence of COVID-19 infection among pre-menopausal women is rare. A gender variance in severe cases infested with SARS-CoV-2 has been reported in China, where approximately 1.7% of women affected are at risk of death compared to 2.8% of men, representing about 50% of the difference [21]. In going in line with this study, Garg et al. showed that the threat of death in men’s population with COVID-19 infection is greater compared to women [14]. Men with SARS-CoV-2 infection are more likely than women to require intensive care unit (ICU) hospitalization [22]. In fact, even if infection incidence by the SAR S–CoV-2 does not seem to differ between the two sexual categories, morbidity and mortality rates are higher in males [21]. Interestingly, previous studies in both humans and animals emphasized sex-specific variances in defenselessness for SARS-CoV [23,24]. Experimental studies on mice infected with SARS-CoV indicated that male mice were more prone to SARS-CoV infection and had higher mortality than females [23]. This evidence along with obtained results from this study, defined a sex variance related with COVID-19 morbidity and mortality. The dissimilarities in vulnerability to viral infection in women and men seem to be reliant on sex hormone production in women [24]. Anti-androgens have the potential to treat the illness because all steroid receptors are expressed in the lung, and altering the activity of steroid receptors may be a key factor in mediating SARS-CoV-2 infections [25]. Additionally, a review finding implied that androgen was involved in the development and severity of COVID-19. This review [22] mentioned the development of COVID-19 treatments based on androgen suppression. Based on obtained results from studies conducted by Seeland et al. [13] and Costeira et al. [15], estrogens possibly play a protective role against COVID-19 infection. Ovarian steroids, especially estrogen at menopause, have been identified to impact the adaptive and innate immune response and reduction of it leads to increased morbidity and mortality of post-menopausal women against viral infection [15]. In this regard, it seems that an increasing degree of mortality and morbidity in COVID-19 postmenopausal women compared to premenopausal in a study conducted by Garg et al. [14] is linked to the defensive role of estrogens in premenopausal females [3].

The renin–angiotensin system (RAS) has extremely declined in SARS-CoV-2 infection, as SARS-CoV-2 binds to ACE2, leading to a considerable downregulation of defensive ACE2 [26] In fact, the SAR S–CoV-2 interacts with the human ACE 2 receptor to enter cells and reduces its expression [26] ACE 2 is widely expressed in numerous tissues and systems such as the lungs, heart, kidneys, gonadal and vessels [27]. ACE2 plays a key role in the transformation of angiotensin I (Ang I) to anti-apoptotic, anti-inflammatory, and antifibrotic products [28]. Therefore, reduction of ACE2 by SARS-CoV-2 leads to initiation of inflammatory and pro-inflammatory activations [26]. Experimental and clinical studies have revealed that the RAS is markedly affected by sex‐related changes [29,30]. The ACE2 gene is required for SARS-CoV-2 to enter host cells [22]. Estrogen may be involved in this outcome since there is data that estrogen has the ability to decrease ACE2 activity [31]. Consequently, it can be postulated that estrogen, through modulating the expression of ACE2, recovers the consequences of the COVID-19 infection by dropping the cytokine storm and inflammation. In addition, experimental and clinical studies propose that estrogens can influence lung inflammation, because intuition of estrogen receptor signaling modifies immune cells, both adaptive and innate immune responses [32,33]. Estrogen receptors are express in immune cells such as neutrophils and macrophages [34] and were identified in resident immune cells and cellular components of the lung [35]. 17β‐estradiol is the main circulating estrogen that, through estrogen receptor activation, can modulate pro‐inflammatory cytokines and decrease the harshness of the inflammation [36]. The positive impacts of estrogens on lung inflammation, immune response, viral infection, and the cardiovascular structure have also been discussed as important factors effecting the progression of disease and the consequences [24,37].

In consistency with the findings of the present review, a review revealed that patients with COVID-19 have lower E2 levels than subjects without COVID-19 [6]. Ding et al. demonstrated that E2 and anti-mullerian hormone (AMH), a member of the transforming growth factors (TGF-P) family and the ideal marker for function and ovarian reserve, have a negative correlation with COVID-19 infection severity [16]. Also, AMH was lower in COVID-19 than in healthy participants of the same age [18]. In a cohort study, users of combined oral contraceptive pills had significantly lower rates of COVID-19 and fewer hospitalizations among affected patients [15]. Another cohort study revealed that the mortality rate of women over the age of 50 who were treated with estradiol was 50% lower than that of women of the same age who did not take the hormone [13]. In line with the cohort studies we included, estrogen supplementation in postmenopausal women was associated with a reduced risk of death from COVID-19 in a nationwide cohort study [38]. These findings suggested that taking oral contraceptives acted as a form of COVID-19 infection protection for E2. In a randomized controlled trial, postmenopausal females who took oral estradiol had a significantly higher rate of reverse PCR negativization, fewer patients who progressed with their disease, and lower Interleukin 6 levels in the intervention group compared to the control group. E2 may lessen the cytokine storm and inflammation associated with COVID-19 disease, as evidenced by the intervention group's significantly different mean values of the inflammatory biomarkers D dimer, LDH, and CRP [19]. According to a study that looked at the effects of treating human lung epithelial cells in vitro with 17-β-estradiol, this hormone may lessen SARS-CoV-2 infection of the cells, which may help to explain why women are less likely than men of similar age to experience severe Covid-19 and fatalities [11]. Furthermore, perimenopausal menopausal hormone therapy's undeniable immunological advantages support the critical function of estrogens in the regulation of immune functionalities [4]. We should exercise caution, however, as higher estradiol was linked to a higher risk of death in both sexes in a cohort study that looked at patients aged 50 and over, despite the fact that raised E2 has been shown to be a novel and effective option for treating non-severe COVID 19 infections [39]. HRT was linked to a decrease in all-cause mortality in COVID-19, according to a cohort of 1,863,478 English women over 18 [40].

There is some evidence that progesterone contributes to gender differences related to COVID-19, even though the majority of studies looked at the potential role of estrogen against COVID-19. Ghandehari et al. [20] in a randomized, controlled pilot trial, demonstrated that progesterone at a dose of 100 mg (b.i.d.) adding to standard of care could represent an effective and safe method for treatment in men hospitalized with confirmed moderate to severe COVID-19. More clinical investigations regarding hormone supplements using AMH and progesterone are needed to validate the therapeutic protective role of these hormones against COVID-19 infection.

On the other hand, testosterone has largely preventative impacts on immune function, which might partially describe the more vulnerability to infections detected in men [41]. It has been postulated that SARS-CoV-2 infection is androgen driven as the expression of the transmembrane protease serine 2 (TMPRSS2), an essential receptor for passing of SARS-CoV-2 onto target cells, is controlled by androgen receptor signaling [41].

The last possible explanation for the gender difference against COVID-19 infection is the presence of numerous genes involved in inflammation and a huge number of immune-related genes accountable for different adaptive and innate immune responses to disease on the X chromosome [42]. Therefore, heterozygous-X women could stimulate advantageous mosaicism and a further noticeable sexual dimorphism compared to the men in the protection against induced pathologies by SAR S–CoV-2 [43]. It should also be mentioned that the ACE 2 gene is placed on the X chromosome and this makes women possibly heterozygous compared to men [43].

One of the weaknesses is that this review included literature published in the English language which might result in losing some studies published in other languages. Notwithstanding a predefined systematic method, the study will also include judgments made by reviewers, which can result in bias. The main limitation of this study was lack of enough clinical trials regarding the protective effect of sex steroid hormones against COVID-19 infection. So, we include 8 studies to understand the concept better of whether sex hormones, gender and menopausal status affect morbidity and mortality of COVID-19 disease. It will be useful to have future clinical trial studies that are completely sex- and age-disaggregated. Several factors might play a role in gender difference against COVID-19, such as related diseases and systemic risk factors. Thus, the recommendation that estrogens might be an ideal protective treatment for COVID-19 has to be taken with caution.

## 5 Conclusion

This study demonstrates a higher mortality rate for men than for women, a higher mortality rate for menopausal women than for young women, and a greater reduction in COVID-19 hospitalization and mortality among women receiving estradiol when viewed as a whole. This study indicates that estrogen may be an effective pharmacological treatment for COVID-19-associated cytokine storm and inflammation reduction. The development of COVID-19 treatments based on estrogen and progesterone has a long way to go, but upcoming clinical trials will provide crucial data that will enhance the available management options. Nonetheless, future prospective and clinical trial studies will be required to define and confirm this protective effect. To further investigate the association between COCP and COVID-19 and the possibility that estrogens may have a protective effect against COVID-19, additional research with larger cohorts is required.

## Author contribution statement

Kowsar Qaderi, Younes Jesmani: Conceived and designed the experiments; Wrote the paper.

Ahmadreza Shamsabadi: Conceived and designed the experiments.

Mehri Kalhor, Marjaneh Pournaghi: Performed the experiments; Wrote the paper.

Maryam Keshavarz: Performed the experiments.

Hossein Hosseinirad, Rasa Khodavirdilou: Analyzed and interpreted the data;Wrote the paper.

Seyedeh Tahereh Mirmolaei: Analyzed and interpreted the data.

Sanaz Zangeneh, Manthar Ali Mallah: Contributed reagents, materials, analysis tools or data; Wrote the paper.

Farideh Eghdampour, Samira Barjasteh: Contributed reagents, materials, analysis tools or data.

## Funding statement

This research did not receive any specific grant from funding agencies in the public, commercial, or not-for-profit sectors.

## Data availability statement

Data will be made available on request.

## Declaration of interest’s statement

The authors declare no conflict of interest.
