# Supplementary material for: The relationship between sex steroids (E2, progesterone, and AMH) levels and severity and fatality of COVID-19: A systematic review
Source: Heliyon. 2023 Mar 1;9(3):e14218. doi: 10.1016/j.heliyon.2023.e14218 (PMC9974207; doi:10.1016/j.heliyon.2023.e14218)
Supplement: Multimedia component 1 [file mmc1.docx]

Key words:

Corona virus, SARS-CoV-2, estrogen, progesterone, "genital hormone"

**Strategy search:**

A: COVID-19 OR SARS-CoV-2 OR “Corona virus” OR Coronavirus OR COVID

B: “genital hormone” OR estrogen OR progesterone OR “follicle-stimulating hormone” OR “Luteinizing hormone” OR “Gonadotropin-releasing hormone”

SEARTH TIME: 2021/10/16

**ISI=33**

**TITLE:** (COVID-19) OR **TITLE:** (SARS-CoV-2) OR **TITLE:** ("Corona virus") OR **TITLE:** (Coronavirus) OR **TITLE:** (COVID) **AND TITLE:** ("genital hormone") OR **TITLE:** (estrogen) OR **TITLE:** (progesterone) OR **TITLE:** ("follicle-stimulating hormone" FSH) OR **TITLE:** ("Luteinizing hormone" LH) OR **TITLE:** ("Gonadotropin-releasing hormone" GnRH)

**SCOPUS= 23**

( ( TITLE ( covid-19 )  OR  TITLE ( sars-cov-2 )  OR  TITLE ( "Corona virus" )  OR  TITLE ( coronavirus )  OR  TITLE ( covid ) ) )  AND  ( ( TITLE ( "genital hormone" )  OR  TITLE ( estrogen )  OR  TITLE ( progesterone )  OR  TITLE ( "follicle-stimulating hormone" )  OR  TITLE ( "Luteinizing hormone" )  OR  TITLE ( "Gonadotropin-releasing hormone" ) ) )  AND  ( LIMIT-TO ( DOCTYPE ,  "ar" )

**PUBMED =24**

(((((COVID-19[Title/Abstract]) OR (SARS-CoV-2[Title/Abstract])) OR (Coronavirus[Title/Abstract])) ) OR (Corona virus[Title/Abstract])) OR (COVID[Title/Abstract]) AND (((((((("genital hormone"[ Title/Abstract]) OR (estrogen[Title/Abstract])) OR (progesterone[Title/Abstract])) OR ("follicle-stimulating hormone"[ Title/Abstract])) OR (FSH[Title/Abstract])) OR ("Luteinizing hormone"[ Title/Abstract])) OR (LH[Title/Abstract])) OR ("Gonadotropin-releasing hormone"[ Title/Abstract])) OR (GnRH[Title/Abstract]) Filters: Full text, Journal Article
